# Patient- and provider-related determinants of generic and specific health-related quality of life of patients with chronic systolic heart failure in primary care: a cross-sectional study

**DOI:** 10.1186/1477-7525-8-98

**Published:** 2010-09-13

**Authors:** Frank Peters-Klimm, Cornelia U Kunz, Gunter Laux, Joachim Szecsenyi, Thomas Müller-Tasch

**Affiliations:** 1 Department of General Practice and Health Services Research, University Hospital Heidelberg, Heidelberg, Germany; 2 Institute of Medical Biometry and Informatics, University Hospital Heidelberg, Germany; 3 Department of Psychosomatic and General Internal Medicine, University Hospital Heidelberg, Heidelberg, Germany

## Abstract

**Background:**

Identifying the determinants of health-related quality of life (HRQOL) in patients with systolic heart failure (CHF) is rare in primary care; studies often lack a defined sample, a comprehensive set of variables and clear HRQOL outcomes. Our aim was to explore the impact of such a set of variables on generic and disease-specific HRQOL.

**Methods:**

In a cross-sectional study, we evaluated data from 318 eligible patients. HRQOL measures used were the SF-36 (Physical/Mental Component Summary, PCS/MCS) and four domains of the KCCQ (Functional status, Quality of life, Self efficacy, Social limitation). Potential determinants (instruments) included socio-demographical variables (age, sex, socio-economic status: SES), clinical (e.g. NYHA class, LVEF, NT-proBNP levels, multimorbidity (CIRS-G)), depression (PHQ-9), behavioural (EHFScBs and prescribing) and provider (e.g. list size of and number. of GPs in practice) variables. We performed linear (mixed) regression modelling accounting for clustering.

**Results:**

Patients were predominantly male (71.4%), had a mean age of 69.0 (SD: 10.4) years, 12.9% had major depression, according to PHQ-9. Across the final regression models, eleven determinants explained 27% to 55% of variance (frequency across models, lowest/highest β): Depression (6×, -0.3/-0.7); age (4×, -0.1/-0.2); multimorbidity (4×, 0.1); list size (2×, -0.2); SES (2×, 0.1/0.2); and each of the following once: no. of GPs per practice, NYHA class, COPD, history of CABG surgery, aldosterone antagonist medication and Self-care (0.1/-0.2/-0.2/0.1/-0.1/-0.2).

**Conclusions:**

HRQOL was determined by a variety of established individual variables. Additionally the presence of multimorbidity burden, behavioural (self-care) and provider determinants may influence clinicians in tailoring care to individual patients and highlight future research priorities.

## Background

Chronic systolic heart failure (CHF) is a common clinical syndrome, with increasing incidence at older age, and is associated with high mortality rates, and compromised health-related quality of life (HRQOL) [1]. Moreover, it is characterised by a high health care utilisation constituting a high burden of disease, mainly due to hospital admissions [1].

The objectives of CHF treatment are to maximise life expectancy, improve HRQOL and prevent disease progression and admissions [2]. Optimal treatment according to clinical practice guidelines [2] and adherence of patients to treatment regimens [3] are paramount. Given the likelihood of poor prognosis, maximising HRQOL is particularly important, especially as a substantial number of patients with CHF prioritize HRQOL over survival [4,5] and patients' perceptions of HRQOL are used increasingly to evaluate the effectiveness of healthcare interventions. Moreover, poorer HRQOL has been shown to be predictive of higher admissions and mortality [6,7].

HRQOL is a multidimensional concept comprised of several domains, including physical/biological factors, symptom status, functional status, health perceptions, and overall well-being [8]. In research, the use of generic and disease-specific instruments to assess HRQOL is recommended [9,10].

Many previous studies have deepened the understanding about what factors can determine HRQOL in CHF, not least to identify intervention targets for improved outcome. They have been performed in various sectors and settings, mostly in secondary care or post-discharge setting [11-22], some in primary care [23-25] and few in the community [26]. Variance of HRQOL has been associated with sociodemographic (e.g. age, gender, socio-economic status [16,18,19,23,25,26]), psychosocial (e.g. depression, anxiety, social support [12,18-21,23-25]), behavioural (e.g. alcohol consumption and smoking [11,25]), clinical (e.g. disease severity assessed by NYHA functional class or peakVO_2_, multimorbidity, BNP [11,13-17,20-25]) and procedural (e.g. vasodilator use [11]) determinants. Heterogeneity of results may be explained by different settings and study designs (e.g. part of a clinical trial or observational study [20,21,25] vs. survey [16,23,24]) leading to heterogeneous samples (e.g. younger patients with systolic HF vs. elderly with HF with preserved systolic function), by different 'availability of variables' and 'use of instruments' for generic vs. disease-specific HRQOL assessment.

Given there is little evidence for patients with CHF recruited in primary care, our aim was to identify and explore the impact of determinants of generic and disease-specific HRQOL with respect to a wide set of individual and provider variables. We focused on an exploratory comparison between generic and disease-specific HRQOL.

## Methods

### Design

This study was conducted as a cross-sectional study of pooled baseline data of *subproject 10 *"quality of life" within the German "Competence Network Heart Failure", sponsored by the Federal Ministry of Education and Research [27]. Within this subproject two primary care-based trials (TTT and HICMan) evaluated different kinds of interventions [28-30]. Both trials conformed to the principles outlined in the Declaration of Helsinki [31] and were approved by the institutional review boards of the local medical faculty of the university and the Medical Association of the federal state Baden-Württemberg in Germany, and were registered (ISRCTN08601529 and 30822978) prior to inclusion of patients.

### GP and patient selection

Interested GPs were eligible for participation if they were certified as a primary care physician or equivalent and practiced as a statutory health insurance affiliated physician. Fifty general practitioners (GPs) from 48 practices participated in the two studies in one region of Northern Baden, Germany.

Eligible patients were adults ≥ 40 years with confirmed systolic heart failure (CHF) with stable symptoms at the time of inclusion, and diagnosis of a chronic, irreversible CHF at least 2 weeks prior to inclusion. CHF diagnosis was based on dyspnea (NYHA) and objective measurement (e.g. by echocardiography) of impaired left ventricular systolic dysfunction (left ventricular ejection fraction ≤ 45%). Criteria slightly differed between the TTT-trial and HICMan-trial regarding the cut-off and actuality of the determination of left ventricular ejection fraction (TTT: ≤ 40% within last 6 months; HICMan: ≤ 45%, within the last 24 months) and dyspnea (TTT: NYHA II-IV; HICMan: NYHA II-IV or NYHA I, if hospital admission because of CHF within the last 24 months). Exclusion criteria were: Primary valvular heart diseases and relevant hemodynamic effects, hypertrophic obstructive/restrictive cardiomyopathy (HOCM/RCM), and people with a concomitant terminal illness, addictive disorders (drug abuse or persisting alcohol abuse despite social, legal or occupational conflicts), dementia or severe psychological illness [28,30].

All GPs and patients gave written informed consent. 367 eligible patients were recruited, 168 within TTT (enrolment of patients and data collection: March to September 2005) and 199 within HICMan (enrolment of patients and data collection: June 2006 to January 2007), details have been described elsewhere [29,30]. Within the HICMan-trial, two patients did not show up after informed consent and 47 patients have participated in the previous TTT-Trial. To obtain baseline data from eligible patients "naive" of (the later tested) interventions, we pooled baseline data of 318 patients (168+150).

### Collection and management of Organisational and Clinical data

GPs received an initiation visit by a study nurse including an introduction to the trial's investigator file. GPs collected and documented organisational (location of practice, list size, no. of GPs per practice, etc.), physicians' individual (e.g. years in practice) and patients' individual clinical data (e.g. history, current clinical status, lab results, ECG, detailed medication etc.) on pre-specified case report forms (CRFs) according to the Basic Clinical Dataset (BCD) of the Competence Network Heart Failure [27]. The documentation of patients' history included single co-occurring medical conditions (such as Angina pectoris, Peripheral Arterial or Cerebrovascular Disease, Hypertension, Diabetes, COPD, Depression etc.. For chronic care in primary care in general, as for patients with CHF [32], the co-existence of multiple diseases is the rule rather than the exception and therefore of special interest. Likewise, there is co-existence of attempts to define the phenomenon of co- and multimorbidity [33-37]: In one classification it has been classified in three cumulative categories: simple co-/multimorbidity (the co-occurrence of diseases, whether coincidental or not); associative co-/multimorbidity (statistical association, not or not known to be causal); and causal co-/multimorbidity (implying a causal relation among co-occurring diseases). Expanded conceptualisations pay attention to „morbidity burden" and „patient complexity" [38]: The former is linked to its impact to patient-centred outcomes such as functioning and is therefore linked to the frailty construct. The latter acknowledges socio-economic, cultural, behavioural and environmental characteristics (see next paragraph). These constructs address different aspects of multimorbidity and are applied in three research areas (clinical care, epidemiology and public health, and health services research [38]. Accordingly, to retrieve an estimate of patients' "morbidity burden" in addition to the documentation of single co-occurring medical conditions, GPs applied the Cumulative Illness Rating Scale (CIRS-G) [39-41]: This index measures the chronic medical illness ("morbidity") burden while taking into consideration the severity of chronic diseases in 14 items representing individual body systems. The final score of the CIRS is the sum of each of the 14 scores, theoretically varying from 0 to 56, a higher score indicating higher impairment. To determine N-terminal Brain Natriuretic Peptide (NT-proBNP), blood was taken separately for the Central Biomaterial Bank, a project of the Competence Network Heart Failure providing a central facility to collect all biomaterial (blood, plasma, serum and DNA) from each patient enrolled in one of the network's clinical trials [42]. NT-proBNP was determined using the Elecsys 2010 Kit from Roche Diagnostics, Germany. The CRFs were sent directly to the responsible Coordination Centre Clinical Trials (CCCT).

### Collection and management of Psychosocial and Behavioural data

Parallel to the clinical baseline assessment, patient-reported questionnaires were handed out by practice personnel.

A sociodemographic dataset was obtained from all patients [43]. For generic health-related quality of life (HRQOL) we used the German version of the Short Form 36 Health Survey (SF-36) [44] and for disease-specific HRQOL we used the German version of the Kansas City Cardiomyopathy Questionnaire (KCCQ) [45], which have been shown to be valid and reliable instruments [45-47]. The SF-36 questionnaire consists of eight dimensions (subscales): Physical functioning, Role functioning (physical), Bodily pain, General health perceptions, Vitality, Social functioning, Role functioning (emotional), and Mental health. SF-36 scores are converted to a (T-) scale of 0 to 100, with higher scores indicating superior health status. Scales are aggregated into two summary measures, the Physical Component Summary (PCS) measure and the Mental Component Summary (MCS) [48]. Empirical research showed that scales that load highest on the PCS are most responsive to treatments that change physical morbidity, whereas scales loading highest on the MCS respond most to drugs and therapies that target mental health [48]. The KCCQ quantifies several health status domains including Physical limitations, Symptoms (stability, frequency, and burden), Self efficacy, (mental) Quality of life, and Social function [45]. To summarise the multiple domains of health status quantified by the KCCQ, a clinical summary score (=Functional status, summarising Physical limitations, Symptom frequency and burden) can be calculated, whereas an overall summary score (KCCQ-os) has been developed that includes Functional status, Quality of life, and Social function domains, with the exception of the domains Symptom stability and Self efficacy. Each scale is transformed to a score of between 0 and 100, with higher scores indicating superior health status. A mean five-point change (or difference) in the scales of the SF-36 [49] and in the KCCQ-os [22,50] are regarded as socially or clinically significant.

Depression was assessed using the German version of the Patient Health Questionnaire depression module (PHQ-9) [51]. It consists of 9 items that each describes one symptom corresponding to one of the 9 DSM-IV diagnostic criteria for major depressive disorder. The use of continuous data in the form of the PHQ-9 summary score (0 to 27 points) indicate depression severity (higher scores indicate higher severity) and a categorical algorithm for major depressive syndrome in accordance with DSM-IV diagnostic criteria can be calculated with favourable diagnostic properties [51-53]. The PHQ-9 compares favourably to other screening instruments, and is recommended for patients with cardiovascular diseases [54].

The European Heart Failure Self-Care Behaviour Scale (EHFScB scale) is a 12-item, self-administered questionnaire regarded as a valid, reliable and practical scale to measure the self-reported self-care behaviour of heart failure patients, for example, daily weighing, fluid restriction, exercise or contacting a health care provider [55]. Scores range from 1-5 (12-60), with low scores implying better self-care behaviour. Patients were asked to return the questionnaires in a pre-specified envelope within seven days. Questionnaires were sent back to the CCCT, where data management was performed [27] - either directly (TTT) or via the study centre (HICMan) to enable the study nurse to monitor the progress of study documentation.

### Procedure and statistical methods

To allow comparisons across the two HRQOL measures we decided a priori to focus on summary measures, i.e. the PCS and MCS (SF-36), and Functional status, (mental) Quality of life, Self efficacy and Social function (KCCQ), all distinct domains that differentiate most adequately between 'physical' and 'psychosocial' aspects of HRQOL. Therefore, we omitted KCCQ-os as it represents aggregated scales of both aspects, rendering a comparison with generic HRQOL (PCS and MCS) difficult.

Our choice of variables to be analysed with respect to their predictive value was based on the literature and clinical reasoning. We selected variables of the provider and individuals respectively as shown in tables 1 and 2.

**Table 1:** Characteristics of 50 general practitioners from 48 practices; values represent number (percentages) of practices unless stated otherwise.

Practice factors	(n = 48)
Location	
rural	25 (52.1)
suburban	10 (20.8)
urban	13 (27.1)

No. of GPs per practice	
One GP	24 (50)
Two GPs	18 (37.5)
> 2 GPs	6 (12.5)

List size (patients per quarter)	
0-999	11 (22.9)
1000-1499	18 (37.5)
≥1500	19 (39.6)

GPs' characteristics	(n = 50)

Mean age of in years (SD) [range]	49.1 (9.0) [33-63]

Female	11 (22)

Practicing as GP since mean years (SD) [range]	15.0 (8.3) [0-33]

Participation in trials (TTT only vs. HICMan only vs. TTT and HICMan)	20/13/17 (40/26/34)

Mean (SD; range) number of patients per GP	6.4 (4.8; 1-18)

GP: General practitioner

Dummy variables were built for all ordinal variables (location of practices, list size and no. of GPs per practice, patients' socio-economic status, left ventricular ejection fraction, Creatinin-Clearance). We aggregated NYHA functional class I with II and II with IV accounting for the low number of observations in the classes I and IV. As NT-proBNP had a skewed distribution, logarithmic transformation was performed as described and performed previously [25,56]. Alcohol consumption (no. of drinks per week) was omitted because of skewed distribution not amenable to transformation.

Pearson correlation coefficients were calculated for analyses of relationships between numerical explanatory variables and the dependent variable. In the following, Creatinin-clearance was omitted due to collinearity with age, which correlated consistently higher with dependent variables.

Variables with a p-value less than 0.05 were entered into multiple regression analyses using the forward selection algorithm. As this procedure uses only those individuals who have complete information on all explanatory variables results were validated in unified regression models. All explanatory variables remained within the models, except for NYHA functional class for PCS and Hypertension for (mental) Quality of life (KCCQ). To account for the clustering of data (intraclass correlation within each practice attributable to clustering) we performed linear mixed effects regression models with the physician as a random effects model nested within groups. Regression coefficients were the same; the few exceptions regarding explanatory variables are reported. Given the possibility to inform about the amount of explained variance (R^2^) we present the results of the unified regression models. For analyses we used Stata/MP version 10.1, SPSS version 16.0.2 (SPSS Inc.) and SAS 9.2 (PROC MIXED).

## Results

### Provider and patient characteristics

Table 1 shows the characteristics of 50 participating GPs, with a mean age of 49.1 (SD: 9) and practicing on average for 15 years (SD: 8.3) years. It also shows the characteristics of the 48 participating practices (location, number of GPs and patients per practice). The mean (SD; range) number of patients per GP was 6.4 (4.8; 1-18).

Table 2 summarises the characteristics of 318 eligible patients regarding socio-demographic, clinical, psychosocial and behavioural variables. Patients were predominantly male (71.4%) with a mean age of 69 (SD: 10.4) years and mostly belonging to the lower or middle social class (36.3% and 42.5%). Most patients were in NYHA functional class II or III (97%) and had a moderately reduced LVEF (35.3 ± 7.2%). In 45% of the cases coronary heart disease was the main cause for CHF as reported by the treating GPs. Mean (SD) duration of CHF was 5.8 (5.1) years. Patients had undergone different cardiovascular interventions, 33% at minimum one PTCA and 22.6% a CABG (coronary artery bypass graft) surgery. Vascular medical conditions were prevalent, e.g. peripheral arterial disease (17.3%), others were hypertension (78.9%), diabetes (36.5%), COPD (23.6%) and Depression (20.8%). Estimated renal function was impaired in more than 40% of cases (GFR < 60 ml/min). Mean (SD) NT-proBNP-levels were 2298.4 (3985.9) pg/ml. Physician-rated mean (SD) multimorbidity as indicated by CIRS-score was 23.8 (5.5). Mean (SD) summary PHQ-9 screening score was 7.2 (5.4), accordingly every 8th patient (12.9%) fulfilled criteria for major depression. Every 7th patient was a smoker; average alcohol consumption was 4.2 drinks per week. Mean (SD) EHFScB scale score (Heart failure self-care behaviour) was 24.7 (7.6).

**Table 2:** Patient characteristics (n = 318); values represent numbers (percentages) of patients unless stated otherwise

Trials source (participation in TTT- vs. HICMan-trial)	168/150 (52.8/47.2)
Sociodemographic characteristics	

Male sex	227 (71.4)

Mean (SD) age (years)	69.0 (10.4)

Social class*:	(n = 277)
lower	117 (36.8)
middle	135 (42.5)
upper	25 (7.9)

Clinical variables	

NYHA-functional class (according to GP)	
I	4 (1.3)
II	185 (58.2)
III	124 (39.0)
IV	5 (1.6)

Mean (SD) LVEF (n = 304)	35.3 (7.2)
LVEF not reported	14 (4.4)
≤ 45%	221 (69.5)
≤ 30%	83 (26.1)

Main cause of CHF	
ischemic	143 (45.0)
non-ischemic	175 (55.0)

Mean (SD) duration (years) of CHF (n = 238)	5.8 (5.1)

Cardiovascular interventions	
PTCA/Stent (any)	105 (33.0)
CABG (any)	72 (22.6)
Pacemaker (right ventricular)	44 (13.8)
Pacemaker (biventricular)	19 (6.0)
ICD	49 (15.4)
Prosthetic heart valve (any)	20 (6.3)
Reanimation/Defibrillation	22 (6.9)

Medical conditions	
Angina pectoris	81 (25.5)
PAD	55 (17.3)
Cerebrovascular disease	60 (18.9)
Hypertension	251 (78.9)
Diabetes mellitus	116 (36.5)
COPD	75 (23.6)
Depression (as rated by GP)	66 (20.8)

Creatinine-Clearance (n = 314): Mean (SD) GFR (ml/min)**	71.2 (31.1)
Stage of renal dysfunction	
GFR ≥ 60 ml/min	182 (57.2)
GFR 30-59 ml/min	119 (37.4)
GFR ≤ 29 ml/min	13 (4.0)

Mean level of NT-pro-BNP*** (SD) in pg/ml (n = 303)	2298.4 (3985.9)

Mean (SD) Comorbidity (CIRS-G)****	23.8 (5.5)

Psychosocial and behavioural characteristics	

Depression (PHQ-9-D)	
Mean (SD) summary score	7.2 (5.4)
Major Depressive Syndrome	41 (12.9)

Mean number (SD) of drinks per week	4.2 (6.4)

Ex-/smoker (Ex: since at least 6 months)	142/46 (44.7/14.5)

Heart failure self-care behaviour (EHFScB scale*****)	24.7 (7.6)

Prescribed drugs	
ACE inhibitor	243 (76.4)
A2RA	62 (19.5)
β-blocker	246 (77.4)
Spironolactone/Eplerenone (Aldosterone- antagonists)	89 (28.0)
Loop diuretics	195 (61.3)

**Social Class according to modified German Winkler-index [43] (lower class: 0-7; middle class: 8-14; upper class: 15-21);

NYHA, New York Heart Association; LVEF, Left ventricular ejection fraction; CHF, Chronic (systolic) heart failure; CHD, Coronary heart disease; PTCA, Percutaneous Transluminal Coronary Angioplasty; CABG, Coronary artery bypass graft surgery; ICD, Implantable cardioverter defibrillator; PAD, Peripheral arterial disease; COPD, Chronic obstructive pulmonary disease;

**Estimation of the GFR according to the formula of Cockroft and Gault;

***N-terminal Brain Natriuretic Peptide;

****CIRS-G, Cumulative illness (physician) rating scale, range 0-56, lower scores imply less impairment of 14 body systems

*****European Self-care Behaviour scale, range 12-60, lower scores imply better self-care behaviour;

ACE = angiotensin converting enzyme; A2RA = angiotensin-2 receptor antagonist

Most patients were treated with ACE inhibitors or angiotensin-2 receptor antagonists, β-blockers and many with loop diuretics. 28% of the patients were prescribed aldosterone antagonists.

### Quality of life

Mean SF-36 scores (subscales and summary measures), KCCQ domains and summary scores are shown in Table 3, which shows that HRQOL was considerably impaired in all SF-36 scales and KCCQ domains.

**Table 3:** Generic and disease-specific mean (SD) quality of life scores of patients

Quality of life measure	Mean values (SD)	No
**SF-36 scales**		

physical functioning	49.0 (28.4)	318

role functioning, physical	38.7 (42.7)	280

bodily pain	61.2 (28.9)	316

general health perceptions	45.9 (19.0)	311

vitality	44.0 (22.5)	308

social functioning	70.6 (27.1)	317

role functioning, emotional	60.6 (46.3)	282

mental health	63.5 (21.9)	305

Physical Component Summary*	36.8 (10.3)	264

Mental Component Summary*	47.0 (11.9)	264

**KCCQ domains**		

physical limitation	62.3 (24.8)	301

symptom stability	49.6 (16.1)	306

Symptoms	68.3 (24.1)	311

Functional status*	65.5 (22.4)	312

Self efficacy*	70.8 (23.0)	310

(Mental) Quality of life*	63.9 (26.0)	311

Social limitation*	63.9 (28.7)	297

Overall clinical summary	64.7 (22.6)	312

*Summary scales and distinct domains in bold represent target variables

### Determinants of health-related quality of life

Table S1 (see Additional file 1) summarises the results of the final six regression models for generic, i.e. the Physical and Mental Component Summary (PCS and MCS), and disease-specific HRQOL, i.e. the KCCQ summary scale Functional status and the distinct domains (mental) Quality of life, Self efficacy and Social limitation (KCCQ). In these models, eleven determinants overall explained between 27% and 55% of variance of HRQOL.

Organisational (provider) variables, i.e. a greater (patient) list size impacted negatively on the MCS and Self efficacy, whereas a higher number of physicians per practice determined MCS positively.

Among sociodemographic factors, age remained in four of the overall six models (PCS, Functional Status, Self efficacy and social limitation; β: -0.14 to -0.21), and a higher socio-economic status in PCS and Self efficacy (β: 0.14 to 0.23).

NYHA functional class contributed only to the explained variance of the disease-specific summary scale Functional Status (β: -0.16), whereas COPD for the generic summary PCS (β: -0.15).

A history of CABG surgery determined the generic HRQOL summary MCS positively (β: 0.09) and the prescription of an aldosterone antagonist the KCCQ domain Social limitation negatively (β: -0.13). Aggregated physician-rated multimorbidity (CIRS-G) contributed to the explained variance in generic and specific scales (PCS, Functional status, (mental) Quality of life and Social limitation; β: -0.09 to -0.14).

Better self-care behaviour (as measured by the EHFScB scale) contributed significantly to the KCCQ domain Self efficacy (β: -0.21).

Depression severity (PHQ-9 summary score) was a significant determinant of generic and disease-specific HRQOL in all models, and had the strongest impact (β: -0.3 to -0.72) on each summary scale of HRQOL in comparison to other determinants.

## Discussion

### Summary of main findings

In a clearly defined convenience sample of outpatients with stable systolic chronic heart failure (CHF), we could explore determinants of generic and disease-specific health-related quality of life (HRQOL) using a wide set of explanatory variables. Generic and disease-specific HRQOL varies but is considerably impaired in these patients. They demonstrated good Heart failure self-care behaviour but a considerable burden of (multi-)morbidity. We explored the impact of sociodemographic variables, objective measures of heart failure severity, somatic and depressive comorbidity, behavioural and provider variables on the variance of generic and disease-specific HRQOL in patients with CHF using multiple linear regression analyses. Eleven determinants were independently associated with generic and/or disease-specific HRQOL: These were depression severity, physician-rated morbidity burden, increasing age, bigger list size, higher NYHA functional class, COPD, prescription of aldosterone antagonist determined worse, whereas more GPs (per practice), a higher socio-economic status, better Self-care and history of CABG surgery determined better HRQOL.

### Findings and their relation to other studies

A considerable number of studies have led to conceptual models of HRQOL in relation to CHF that describe the interactive relationships between pathophysiology, symptoms (e.g. dyspnea, fatigue, ankle swelling), functional and psychological aspects [57]. According to Rector, people with CHF need to perceive symptoms - abnormal states produced by the pathophysiology - before their HRQOL is affected by CHF, either directly or indirectly. The model acknowledges further the influence of other exogenous factors, such as personality traits, lifestyle demands, culture and multimorbidity that might alter the apparent relationships.

Our results regarding the impact of objective measures such as EF and BNP on HRQOL are similar to previous literature: For example, while BNP correlated bivariately with HRQOL scales, it did not remain in the multivariate regression analyses with included (known) correlates such as NYHA functional class. Therefore, decreased EF or elevated BNP seems not to be sensed by the individual, but the associated symptoms or functional status [13,17].

Depression severity as assessed by the summary score of the PHQ-9 had by far the greatest impact on HRQOL variance in all six investigated summary scores or domains, a finding that is in line with previous findings: Gott et al. found depression, measured by a geriatric depression scale, determining generic and specific HRQOL (summary scores of SF-36 and KCCQ) in a cross-sectional study with 542 elderly (mean age 77 years) patients in primary care, a sample where the diagnosis of CHF was validated by the GP [23]. In our own previous study, we found depression severity (measured by PHQ-9) to be by far the strongest determinant of subscales of SF-36 in a primary care-based sample of 167 patients (mean age 68 years) with ascertained systolic HF [25].

In this study, NYHA functional class determined KCCQ Functional Status, but not the Physical Component summary of the SF-36. There, COPD, a disease characterised also by 'dyspnea' was independently associated. These findings are supportive for the higher specificity of the KCCQ than the SF-36 with regard to the cardinal symptoms of CHF.

Moreover, our results regarding the role of disease severity (NYHA class) and Depression (PHQ-9 summary score) in disease-specific HRQOL (KCCQ) are in line with Faller et al. [20]. They investigated the impact of disease severity (represented by NYHA class) and depression (represented by the categorical algorithm for minor or major depression for the PHQ-9 score) in a sample of 233 heart failure outpatients of a university hospital (mean EF 43%, NYHA I/II/III in 15.9/39.5/34.8% of patients). Disease severity and Depression impacted on the full range of KCCQ domains and summary scores, while they found significant interaction in the KCCQ domain (mental) Quality of life. The authors discussed confounding due to the structural overlap between the PHQ-9 and the quality of life domain of the KCCQ, and biased patients' perception in the sense of over-reporting of subjective symptoms. In a consecutive study entailing 206 subjects from the same sample, by using structural equation techniques, Faller et al. could determine the independent extent of impact of disease severity and Depression on the domains (mental) Quality of life and Physical limitation. They found that depression influenced not only the psychological (ß = 0.75), but also the physical domain (ß = 0.3), whereas heart failure severity, as measured by NYHA functional class, affected the physical (ß = 0.44), but merely the psychological (ß = 0.12) domain. Our results are at the least coherent with these findings, as depression severity impacted on KCCQ Functional status and (mental) Quality of life, but heart failure severity (NYHA functional class) only on Functional status, but not (mental) Quality of life, even though Faller et al. chose the KCCQ domain Physical limitation and used structural equation techniques, which is more appropriate as it allows for simultaneous analysis of the impact of multiple explanatory variables on several dependent variables, which was not the focus of our study.

Notably, physician-rated overall morbidity burden (multimorbidity) was considerable (CIRS summary score) and determined HRQOL in 4 of 6 investigated models, i.e. in the PCS of the SF-36, but not MCS, and in all KCCQ models, except for Self efficacy. Studies that investigate co-/multi-morbidity in relation to HRQOL of patients with CHF usually account for single diseases and/or count the no. of conditions and rarely used the CIRS instrument: We included the CIRS additionally to certain single conditions as a physician-rated disease severity aggregate accounting for all body systems would better reflect the patients' disease burden. Regarding generic HRQOL, our results are consistent to a study in primary care with a sample of 238 patients with chronic diseases: The CIRS played a role within the PCS (R^2 ^0.18), but not within the MCS [58] and is in line with empirical research that showed that scales that load highest on the PCS are most responsive to treatments that change physical morbidity, whereas scales loading highest on the MCS respond most to drugs and therapies that target mental health [48].

In a study with patients with CHF, the CIRS score explained only a small part of the variance in one subscale of the SF-36 - Bodily pain [25]. In this study, no summary measures were analysed rendering a comparison across levels of aggregation difficult, but the different extent of impact of multimorbidity on generic HRQOL is striking. Gott et al. could show the negative impact of multimorbidity on generic and disease-specific HRQOL, but multimorbidity was measured by counting the number of conditions [23]. The impact of the comorbidity COPD on PCS is congruent to Müller-Tasch et al. and Franzén et al. who also found an impact of respiratory diseases on the physical dimensions of HRQOL [16,25]. The finding of history of CABG surgery and its impact on the MCS of the SF-36 is difficult to interpret and should be replicated by further studies. One might argue that a definitive therapy with improved patient outcome regarding symptoms (Angina pectoris) might impact also on generic aspects of HRQOL. However, pectoral angina was not a significant correlate in our study. We conclude that the role found for multimorbidity (measured by CIRS) represents appropriately the perspective of primary care, where patients suffer from more than one index disease, and balancing care and treatment together with the patient is crucial. The CIRS measure seems the best available for primary care [59], and an electronic version of the instrument provides a practical application either for clinical or research use [60], but future studies need to clarify its value regarding HRQOL and prognosis in general practice [61,62].

Socio-economic status (SES) impacted on KCCQ Self efficacy and SF-36 PCS. Little is known regarding this aspect in relation to CHF. In the study of Gott et al. lower SES impacted negatively on MCS and KCCQ overall scores [23], and education determined aspects of HRQOL [11,15,26] or compliance [63], in patients with heart failure. There is general knowledge that social inequalities are associated with morbidity and mortality [64,65], and also with health behaviour [66]. Our results may add another additional finding that higher educated people (SES) have lower levels of emotional and physical distress, reduced by way of paid work and economic resources, which are associated with high personal control [67].

A larger practice list size was associated with worse HRQOL (MCS and Self efficacy), whereas a higher number of GPs per practice counterbalanced this observed association (in MCS), which is a new finding, as there is little evidence for the impact of organizational aspects, i.e. practice factors, on HRQOL in CHF in routine care. Moreover, our variables on organization (e.g. workload, full-time equivalents, skill mix, degree of delegation, use of chronic care services) were not comprehensive.

However, some literature on practice performance and patient satisfaction shows associations within the organization of primary care that cannot be fully explained [68-70]: In a cross-sectional study of 1188 general practices in The Netherlands, large practices showed no clear association with higher assistant volumes and GPs' workload; large practices had lower assistant volumes, but more chronic care services [68]. In an observational study of 140 practices across Europe [69], a larger practice size was associated with lower GPs' workload, but not chronic care organisation (according to the Chronic Care Model). In a further study in 239 Dutch general practices [70], van den Hombergh et al. found that GPs providing more care time in the practice, and more time per patient and experiencing less job stress are all associated with patients' perceptions of better care and better practice performance. In the context of these results, our finding would make sense. Thus, it warrants consideration as a potential determinant of HRQOL, while at the same time it needs to be replicated in future studies together with a more complete set of other explaining organisational variables.

The EHFScBS scores that aggregate the actual patient-reported self-care behaviour (12 items) were associated with KCCQ Self efficacy (2 items), as expected, although the two instruments ask not completely the same concepts: The instruments differ in that the EHFScBS asks for patient's agreement on defined 12 behaviours, and the KCCQ Self efficacy asks about patient's *sureness *on what to do if heart failure worsens and about the patient's *understanding *of the ability to prevent worsening of CHF (for example, weighing yourself, eating a low salt diet, etc.). According to the European Clinical Practice Guideline for CHF, HF self care can be defined as *action *aimed at clinical stability, avoidance of behaviour that can worsen the condition, and early detection of symptoms of deterioration. Self care management is regarded as a key issue of successful treatment and can significantly impact on health outcomes [2]. According to the author of a systematic review on self-care and HRQOL in patients with CHF, findings from RCTs of self-care, as an intervention, on HF patient HRQOL do not allow strong conclusions about the benefits because of methodological and conceptual issues [71]. The author advocated large multi-site RCTs with self-care as the primary intervention.

### Limitations of the study

A number of limitations should be stated: The cross-sectional design of our study implies that no cause-effect relationship between variables can be established. The explorative approach rather implies the identification of independent associations, whereas predictive value needs to be validated in consecutive studies. A known dilemma exists between internal validity and generalisability: Participants of the parental trials might not be representative for the population, e.g. because elderly patients may decline to participate because of effort. On the other hand, it can be regarded as strength - contrary to most other studies in primary care - the sample consisted of patients with defined CHF, so more valid statements can be made regarding this patient group.

Most complete models were obtained for KCCQ Functional Status and (mental) Quality of life, whereas most missings pertained to SF-36 summary scores, socio-economic status and self-care. We abstained from imputation methods as they have their own limitations. We did not perform a non-responder analysis, but regard our approach in the context of exploration as appropriate.

## Conclusions

Considerably impaired and varying health-related Quality of life (HRQOL) of patients with systolic heart failure in primary care could be explained by known determinants in different patterns and to various extents across generic and disease-specific concepts of HRQOL, such as age, social status, depression symptoms and disease severity. Patients' perceptions of disease severity (depression and functional decline) were the strongest determinants, whereas, physician-rated multimorbidity (morbidity burden) also impacts independently on HRQOL - more than findings in previous studies suggest. New potentially relevant variables, i.e. organisational aspects of primary care, need to be confirmed in future studies. Our results also corroborate the ongoing challenge of holistic care for elderly (multimorbid) patients (with CHF) in primary care. Information about patient and provider-related determinants of HRQOL in patients with CHF may help in providing individually tailored care.

## Abbreviations

A2RA: Angiotensin-2 receptor antagonist; ACE: Angiotensin converting enzyme; CCCT: Coordination Centre Clinical Trials; CABG: Coronary artery bypass graft surgery; CHD: Coronary heart disease; CHF: Chronic (systolic) heart failure; CI: Confidence interval; CIRS-G: Cumulative illness rating scale, geriatric; COPD: Chronic obstructive pulmonary disease; EHFScBS: European Heart Failure Self-care Behaviour Scale; GFR: Glomerular filtration rate; GP: General practitioner; HICMan: Heidelberg Integrated Case management; HRQOL: Health-related quality of life; ICD: Implantable cardioverter defibrillator; KCCQ: Kansas City Cardiomyopathy Questionnaire; LVEF: Left ventricular ejection fraction; LVSD: Left ventricular systolic dysfunction; NT-proBNP: N-terminal Brain Natriuretic Peptide; NYHA: New York Heart Association; MCS: Mental Component Summary (of SF-36); PAD: Peripheral arterial disease; PCS: Physical Component Summary (of SF-36); PHQ-9: Depression module of the Patient Health Questionnaire; PTCA: Percutaneous Transluminal Coronary Angioplasty; RCT: Randomised controlled trial; SF-36: MOS 36-item short-form health survey; TTT: Train the trainer (-trial)

## Competing interests

The authors declare that they have no competing interests.

## Authors' contributions

FPK, CUK and TMT designed the study. CUK, GL and FPK analysed the results. All authors interpreted the results. FPK wrote the manuscript, and all authors contributed to writing revisions and approved the final manuscript. FPK is the guarantor.

## Supplementary Material

Additional file 1 **Table S1:** Determinants of generic (SF-36) and disease-specific (KCCQ) health-related quality of life (HRQOL) Click here for file
