# Supplementary material for: Patient- and provider-related determinants of generic and specific health-related quality of life of patients with chronic systolic heart failure in primary care: a cross-sectional study
Source: Health Qual Life Outcomes. 2010 Sep 13;8:98. doi: 10.1186/1477-7525-8-98 (PMC2945966; doi:10.1186/1477-7525-8-98)
Supplement: Additional file 1 — Table S1: Determinants of generic (SF-36) and disease-specific (KCCQ) health-related quality of life (HRQOL) [file 1477-7525-8-98-S1.DOC]

Table S1: Determinants of generic (SF-36) and disease-specific (KCCQ) health-related quality of life (HRQOL)

|  | **Linear Regression Models for distinct HRQOL scores (ß, non-standardised regression coefficient (95%CI))** | | | | | |
| --- | --- | --- | --- | --- | --- | --- |
|  | **SF-36** | | **KCCQ** | | | |
|  | **Physical Component Summary** | **Mental Component Summary** | **Functional status** | **(Mental) Quality of life** | **Self efficacy** | **Social limitation** |
| **No of observations** | 232 | 258 | 302 | 301 | 239 | 289 |
| **R^2^ for the model (adjusted)** | 34.7 (33.2) | 55.9 (55.2) | 50.4 (49.8) | 47.1 (46.1) | 29.4 (27.3) | 46.0 (45.3) |
| **Practice factors (List size/No. of GPs)** |  |  |  |  |  |  |
| **1000-1499 patients**  **≥ 1500 patients** |  | -0.15, -3.5 (-5.6; -1.5)** ^a^ |  |  | -0.16, -7.7 (-13.0, -2.5)** |  |
| **2 GPs**  **> 2 GPs** |  | 0.10, 4.0 (0.5; 7.6)* ^b^ |  |  | -0.18, -8.1 (-13.2, -3.1)** ^d^ |  |
| **Sociodemographic factors** |  |  |  |  |  |  |
| **Age** | -0.20, -0.21 (-0.3;-0.1)*** |  | -0.21, -0.5 (-0.6, -0.3)*** |  | -0.16, -0.4 (-0.6; -0.1)** | -0.14, -0.4 (-0.6,; -0.1)** |
| **Social class**  **upper**  **middle** | 0.12, 4.3 (0.2; 8.4)* |  |  |  | 0.23, 18.4 (9.1; 27.8)***  0.14, 6.4 (1.2; 11.7)* |  |
| **Clinical factors** |  |  |  |  |  |  |
| **NYHA functional class** |  |  | -0.16, -7.1 (-11.1, -3.0)** |  |  |  |
| **COPD** | -0.15, -3.7 (-6.4;-0.9)** |  |  |  |  |  |
| **History of CABG surgery** |  | 0.09, 2.5 (0.2; 4.9)* |  |  |  |  |
| **Multimorbidity (CIRS-G)** | -0.14, -0.2 (-0.5;-0.04)* |  | -0.09, -0.4 (-0.7; 0.03)^c^ | -0.12, -0.6 (-1.0, -0.2)** |  | -0.12, -0.7 (-1.2 ; -0.2)** |
| **Aldosterone-antagonist** |  |  |  |  |  | -0.13, -8.2 (-13.7; -2.7)** |
| **Self-Care (EHFScB scale)** |  |  |  |  | -0.21, -0.6 (-0.9; -0.3)*** |  |
| **Depression (PHQ-9)** | -0.41, -0.8 (-1.0; -0.6)*** | -0.72; -1.6 (-1.8; -1.4)*** | -0.60, -2.5 (-2.8; -2.1)*** | -0.64, -3.1 (-3.5; -2.7)*** | -0.30, -1.3 (-1.8; -0.8)*** | -0.61, -3.2 (-3.7; -2.7)*** |

*p<0.05; **p<0.01; ***p<0.001;

^a, b, c, d^ Non-standardised regression coefficient(s) [p-value] in the PROC MIXED model accounting for cluster effect:

^a^ for small practices (<1000 patients) 5.3 [0.003], for middle-sized practices (1000-1499 patients) 3.4 [0.01], against practices with a big list size (≥ 1500 patients) (=0)

^b^ for single practices (one GP) -5.1 [0.02], for practices with two GPs -3.4 [0.08], against practices with more than 2 GPs (=0)

^c^ -0.45 [0.023]

^d^ Structure variable (no. of GPs) no more significant
